# Mitochondrial RNA Helicases: Key Players in the Regulation of Plant Organellar RNA Splicing and Gene Expression

**DOI:** 10.3390/ijms25105502

**Published:** 2024-05-17

**Authors:** Ron Mizrahi, Oren Ostersetzer-Biran

**Affiliations:** Department of Plant and Environmental Sciences, The Hebrew University of Jerusalem, Edmond J. Safra Campus—Givat Ram, Jerusalem 9190401, Israel

**Keywords:** plant, mitochondria, RNA helicase, RNA binding protein, post-transcription, RNA metabolism, splicing

## Abstract

Mitochondrial genomes of land plants are large and exhibit a complex mode of gene organization and expression, particularly at the post-transcriptional level. The primary organellar transcripts in plants undergo extensive maturation steps, including *endo*- and/or *exo*-nucleolytic cleavage, RNA-base modifications (mostly C-to-U deaminations) and both ‘*cis*’- and ‘*trans*’-splicing events. These essential processing steps rely on the activities of a large set of nuclear-encoded factors. RNA helicases serve as key players in RNA metabolism, participating in the regulation of transcription, mRNA processing and translation. They unwind RNA secondary structures and facilitate the formation of ribonucleoprotein complexes crucial for various stages of gene expression. Furthermore, RNA helicases are involved in RNA metabolism by modulating pre-mRNA maturation, transport and degradation processes. These enzymes are, therefore, pivotal in RNA quality-control mechanisms, ensuring the fidelity and efficiency of RNA processing and turnover in plant mitochondria. This review summarizes the significant roles played by helicases in regulating the highly dynamic processes of mitochondrial transcription, RNA processing and translation in plants. We further discuss recent advancements in understanding how dysregulation of mitochondrial RNA helicases affects the splicing of organellar genes, leading to respiratory dysfunctions, and consequently, altered growth, development and physiology of land plants.

## 1. The Regulation of Post-Transcriptional RNA Processing in Land Plant Mitochondria

Mitochondria serve as the powerhouse of eukaryotic cells. Accordingly, these organelles play a central role in cellular energy production through the oxidative phosphorylation (OXPHOS) pathway. As descendants of a free-living bacterium, mitochondria contain their own genetic material (mtDNA, or mitogenome), which encodes tRNAs, rRNAs and some key proteins of the respiratory system. The vast majority of the proteins that reside within the mitochondria are encoded by nuclear gene-loci and are post-translationally imported into the organelles. These include proteins of the OXPHOS system, as well as essential factors that regulate the mtDNA replication, organellar gene expression and the assembly of organellar complexes [1,2,3]. The analyses of the genome sequences and transcriptome landscapes of mitochondria provided key insights into the evolution of mitogenomes in animals, fungi and plants, in addition to the unique properties of mitochondrial RNA (mt-RNA) metabolism, transcriptional activities and protein synthesis in different organisms. Despite all mitochondria having a common bacterial ancestor, they have undergone divergent evolution in different organisms.

The mitochondria in animals typically exhibit a compact genome structure (harboring 37 genes, with 13 protein ORFs), which is mainly transcribed from two initiation sites, resulting in two long polycistronic pre-RNAs, encoded by the light (L) and heavy (H) strands. Post-transcriptional cleavage events generate ‘individual’ mRNA molecules, which are translated into the mitochondrial proteins. Additionally, tRNAs and rRNAs, which are essential for mitochondrial protein synthesis, are also processed from the precursor organellar molecules. The mitogenomes of land plants are notably larger and more complex in structure than their counterparts in Animalia, with a highly sophisticated mode of organellar gene expression at the post-transcriptional level [1]. To become functional RNAs (mature tRNAs, rRNAs and mRNAs), the precursor (pre-) RNAs that are transcribed within the mitochondria in plants must undergo a series of maturation steps. These include the processing of both 5′ and 3′ termini, numerous RNA ‘editing’ events (typically C-to-U deamination reactions in angiosperms’ mitochondria) and the removal of a large number of introns (mostly group II-type) which reside within the coding regions of many essential genes (e.g., respiratory proteins, cytochrome c maturation (CCM) factors, as well as ribosomal subunits). These RNA processing steps are crucial for plant organellar gene expression, and hence for the proper functioning of the respiratory apparatus, ATP synthesis and optimal plant growth and development.

Notably, the intricate interplay of different molecular machineries within the mitochondria is pivotal for the biogenesis of the respiratory machinery and, consequently, for maintaining cellular homeostasis and plant physiology. Among these components, RNA binding proteins (RBPs), which are encoded by nuclear gene loci and imported post-translationally into the organelle, stand out as key players in mitochondrial gene expression [2,4,5]. Furthermore, the organellar-localized RBPs are also anticipated to serve as a key control mechanism for the regulation of mitochondrial biogenesis, the assembly of the OXPHOS complexes and optimal respiratory activities. Here, we focus on the functional and structural aspects of RNA helicases, an ancient group of enzymes, and discuss their significance in modulating mt-RNA splicing, and hence the fidelity of gene expression in land plant’s mitochondria.

## 2. RNA Helicases as Key Players in Organellar RNA Metabolism

RNA molecules, as their highly versatile proteinaceous counterparts, need to adopt a specific molecular conformation and interact with protein cofactors in order to function correctly [6,7,8,9,10]. A cellular challenge is that unlike (most) proteins, RNAs can adopt different non-functional conformations that have similarly stable energetic states, resulting from their chemical simplicity (i.e., combinations of ribonucleotides that are joined together by phosphodiester bonds) [11]. Specific changes in the RNA conformation can be facilitated by a variety of cellular factors, such as RNA binding proteins (RBPs), ligands and metal ions. Additionally, they can be influenced by variations in salt concentration, temperature or pH [12]. This review focuses on RNA helicases as central regulators in the splicing and processing of introns (mostly group II-type intron RNAs) in the genomes of plant mitochondria.

**Figure 1:**
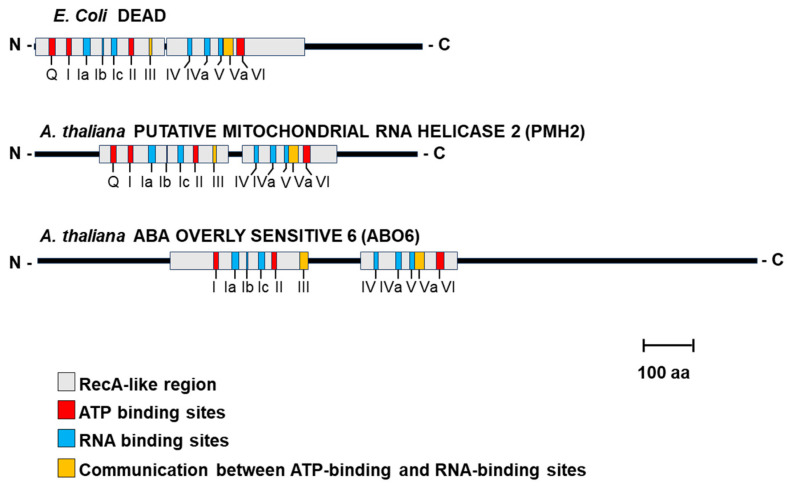
Schematic illustration of functional helicase domains. The canonical core domains and the sub-helicase motifs found within the model DEAD-box helicase DEAD in *E. coli*, as well as two DExD/H-box helicases from *Arabidopsis thaliana* (i.e., the DEAD-box PUTATIVE MITOCHONDRIAL RNA HELICASE 2/PMH2 and the DExH box ABA OVERLY SENSITIVE 6/ABO6) are illustrated. The different sub-motifs (i.e., Q, I, Ia, Ib, Ic, II, III, IV, IVa, V, Va, VI) composing the two RecA-like regions of the DExD/H box proteins are highlighted in light grey. Different colors represent the different biochemical functions of each sub-motif. Red indicates ATP binding sites; blue color represents RNA binding sites; yellow color indicates linker regions between ATP- and RNA-binding sites. DExH and DEAH box proteins lack the Q sub-motif found in DEAD-box helicases.

RNA helicases are a diverse family of enzymes that play key roles in determining RNA secondary (and tertiary) structures, often in an ATP-dependent manner [13]. These also form one of the largest groups of enzymes in eukaryotic RNA metabolism [14]. RNA helicases (and RNA helicase-like proteins) are present in the three kingdoms of life, as well as in the genomes of viruses, and are generally classified into six main groups [15]. The majority of the RNA helicases are clustered with the Super Family 2 (SF2) enzymes [16], which includes the DEAD, DEAH and DExH protein subfamilies. The core motif of the more common DEAD-box RNA helicases consists of two sequential RecA-like domains, which harbor several (i.e., ≥12) well-conserved amino acid subdomains termed Q, I, II, III, IV, V and VI [16]. Figure 1 shows a characteristic structure of DEAD-box RNA helicase proteins.

**Figure 2:**
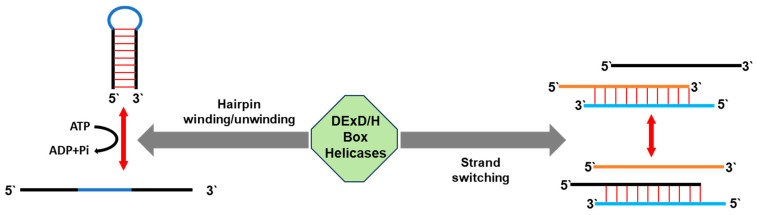
RNA helicases as mediators of RNA structure remodeling. RNA helicases are primarily associated with rearranging RNA structures activities, mostly by unwinding double-stranded RNAs (dsRNAs) into single-stranded RNAs (ssRNAs) (i.e., hairpin winding/unwinding). The combined activities of annealing and unwinding of RNAs promote the exchange of RNA strands (strand switching). These complex processes rely on ATP hydrolysis, not solely for structural rearrangement, but also for facilitating the association of the helicase with the RNA substrate. Accordingly, the schematic figure of the DExD/H box helicases provides insights into the dynamic mechanisms by which RNA helicases remodel RNA structures, highlighting the dual functions of unwinding and annealing, as well as the vital process of ATP hydrolysis in helicase–RNA substrate interactions.

The breaking of hydrogen bonds between ribonucleotides facilitates changes in RNA structures, thereby affecting the stability and function of various RNAs. By unwinding the secondary structures of organellar transcripts, proteinaceous factors, such as RNA helicases, regulate the processing of tRNAs, rRNAs and mRNAs, control the stability of the RNA ligands, resolve RNA–RNA interactions and modulate RNA–protein complexes (Figure 2). These activities are pivotal in numerous key processes, such as in transcription, post-transcriptional processing (such as in splicing), RNA decay and translation (e.g., where the RNA fold may impede ribosome movement) [2,3,17]. Likewise, RNA helicases play key roles in the regulation of RNA metabolism in land plants, and may also link gene expression patterns with developmental or environmental signals [18]. Indeed, several RNA helicases were shown to respond to environmental signals and were also associated with cellular response to abiotic stresses in prokaryotes [15]. Similarly, changes in temperature (or other environmental factors) can affect the expression and/or activity of the organellar RNA helicases, influencing the overall RNA metabolism within the mitochondria (or plastids) [4]. It is, therefore, not surprising that mutations in genes encoding RNA helicases can lead to notable organellar dysfunctions, highlighting the importance of these enzymes for cellular activities and (plant) physiology [2,3,19].

Accumulating data indicate that RNA helicases often act in multi-proteinaceous complexes, and affect various aspects of RNA metabolism and gene expression, e.g., translation initiation assemblies, or within the RNA splicing complexes [15]. Besides canonical helicase motifs, the RNA helicases’ deduced protein sequences often feature additional N- and/or C-terminal domains, primarily comprising protein-protein interaction sites. However, these domains may also confer specific RNA ligand binding capabilities. We elaborate here on the established functions of RNA helicases in the regulation of group II introns splicing in plant mitochondria.

## 3. Plant Mitochondrial Intron Splicing Relies on Nuclear RNA Cofactors

Splicing is a vital processing step in the maturation of some organellar transcripts in the mitochondria of fungi and in plant organelles. This intriguing RNA processing step has arisen as a fundamental process in the regulation of gene expression in the nuclear genomes of eukaryotic cells [20,21,22,23,24]. The splicing reaction entails the removal of intervening RNA sequences (i.e., introns) to allow the joining of the flanking ‘exons’, so as to generate functional RNA (mature, mRNA) transcripts. The introns in nature are divided into several distinct classes, termed group I, group II, group III and nuclear-spliceosomal introns [25]. Each class represents a unique category of intronic sequences with distinct characteristics and functions. These classes serve as fundamental components of genetic information processing across various organisms, playing crucial roles in gene expression and regulation, and in genome evolution. Canonical group I, group II and group III introns are capable of self-splicing, generally under non-physiological (high temperature and salt) conditions. Group I introns are removed from the pre-RNA with a nucleophilic attack by a free nucleotide, usually a free guanine (G) residue. Group II sequences are spliced with a mechanism identical to the nuclear spliceosome, i.e., two transesterification reactions and the excision of the intron as a lariat loop. Following transcription, the group II RNAs can fold into a secondary and tertiary structure consisting of several domains. Group I RNAs fold into nine paired regions (i.e., P1-P9), which are organized into two main domains. Group II RNAs fold into a six-domain secondary structure (DI-DVI). Both group I and group II introns harbor an open reading frame (Intron Encoded Protein, IEP, or maturase) which specifically binds to its own cognate intron and assists with its splicing under physiological conditions, in vivo. In group II introns, the maturases/IEPs are encoded within the fourth domain (DIV).

The organellar introns in plants, fungi and some protists, belong mainly to group I and group II introns [26,27,28,29,30,31,32]. Group II introns are predominantly prevalent in the mtDNAs of vascular plants [33]. One hypothesis suggests that the spliceosome, along with the intron-exon structure of genes, originated from ancient catalytic group II-type ribozymes. According to this hypothesis, the group II intron ribozymes, along with their maturase (intron-encoded) factors, have invaded the eukaryotic genomes during the endosymbiosis of an α-proteobacterium, which ultimately led to the establishment of mitochondria [20,21,22,23,24]. The catalytic and mobile intron RNAs then ‘infected’ and spread through the eukaryotic genome. They gradually degenerated, losing their self-splicing activities, and instead acquired protein cofactors to assist with their splicing in vivo. The nuclear introns and their cognate splicing cofactors later became part of the general splicing machinery, known as the spliceosome. Yet why splicing has expanded in plant mitochondria, while it has been lost in the organelles of mammals (and most other animals), remains an intriguing subject in evolutionary biology [4,21,22,26,34].

While the splicing of nuclear introns is a well-studied process, the cellular mechanisms and factors that enable the excision of the plant mitochondrial group II introns are much less understood [2,21,23,33]. Genetic and biochemical analysis led to the characterization of many proteinaceous cofactors that function in the processing of mitochondrial group II introns in fungi and plants [3,19,35,36]. The majority of the organellar splicing factors are encoded in the nucleus, thus acting as mediators of communication between the nucleus and the organelles [21]. These include a few proteins related to intron-encoded maturases (MATs, Pfam-01348; [37]) that have translocated from the mitochondria into the nucleus [21,38]. Additionally, the majority of the organellar splicing factors in plants include nuclear-encoded proteins that harbor RNA binding modules, such as the pentatricopeptide motif (PPR, Pfam-13812; [39]), the mitochondrial transcription termination factor domain, (mTERFs, Pfam-02536; [40,41,42]), the plant organelle RNA recognition motif (PORR, Pfam-11955), as well as the ubiquitous DExD/H box RNA helicases (Pfam-00270; [3,19,35]) (Figure 1) that are highly conserved in nature and participate in nearly all aspects of RNA metabolism [14]. Table 1 summarizes a list of various DEAD-box RNA helicases found in the organelles of yeast and Arabidopsis, some of which were shown to act in the splicing of group II introns found in the mitogenomes of fungi and land plants.

## 4. Mitochondrial RNA Helicases Factors in *Arabidopsis thaliana* Plants

RNA helicases play pivotal roles in mtRNA turnover, splicing and translation, as well as in the maintenance of the mitochondrial genome in humans, fungi and plants. These were extensively studied in the context of group II intron splicing, especially in yeast mitochondria. A few mitochondrially localized RNA helicases have been described in *S. cerevisiae*, IRC3, MRH4, MSS116 and SUV3. Mutants in these genes show mtDNA defects and exhibit a *petite* mutant phenotype (i.e., formation of small anaerobic-like colonies) [59]. The *petite* phenotype is tightly correlated with altered respiratory functions. mtDNA maintenance can directly rely on the activities of RNA helicases, or might be indirectly influenced by variations in mitochondria gene expression. mtDNA copy numbers were shown to be regulated by both internal and external stimuli. Proteins that control mtDNA replication can also indirectly affect organellar gene expression, by increasing the number of gene-loci that are being actively expressed. Correspondingly, altered mtDNA expression (in the transcriptional, post-transcriptional or translational levels) may also affect the maintenance or stability of the mtDNA [60,61], a phenomenon that is probably related to the effects of partially assembled respiratory complexes, e.g., as indicated in the case of ATP synthase enzyme assembly intermediates [62,63]. Hence, the loss, or a partial reduction, in mtDNA copy numbers may allow the cells to survive under conditions that disrupt the assembly of enzymes of the OXPHOS pathway [64].

The IRC3 enzyme contains a DEAH tetrapeptide within its RNA helicase motif II (Figure 2), but the deduced protein sequence seems to be closely related to the DEAD subfamily [65]. IRC3 was identified in a genetic screen for genes required for respiratory growth, mtDNA maintenance and mitochondrial protein synthesis in *Saccharomyces cerevisiae* [66], and was mainly associated with mitogenome maintenance or replication [64]. The specific roles of IRC3 in mtDNA maintenance, RNA metabolism and or organellar functions are still being investigated. Genetic analyses indicate that the loss of IRC3 results in mtDNA fragmentation [43]. However, as indicated above, genetic analyses showed that the mtDNA defect phenotypes might be indirectly related to altered mitochondrial gene expression or RNA metabolism defects in the yeast cells [60].

Another member of the mitochondrial DEAD-box helicases of *S. cerevisiae* is MRH4, whose loss also results in a *petite* mutant phenotype and altered mitogenome stability [67]. The *MRH4* gene was identified in a genetic screen of nuclear genes that assist splicing of mitochondrial localized group II introns in yeast [67]. The MRH4 was originally defined as a suppressor of a splicing defect linked to a point mutation in the ai5γ intron [67]. However, the main function(s) of the protein seems to be rather associated with mitochondrial translation and/or ribosome biogenesis [64]. Accordingly, null *mrh4* mutants display defects in the assembly of the mitochondrial large ribosomal subunit (LSU), and the MRH4 is suggested to play key roles in the late stages of the mitoribosome assembly by ‘promoting remodeling of the 21S rRNA-protein interactions’ [44].

The *SUV3* gene locus (Table 1) encodes an extensively studied RNA helicase that belongs to the DExH subfamily and harbors an atypical D-E-I-Q sequence within the helicase motif II (Figure 2) [46,47]. Genetic and biochemical studies indicate that SUV3 is a component of the mitochondrial degradosome, and acts as a 3′ to 5′ exonuclease complex together with the DSS1 ribonuclease [47,68,69,70,71,72]. These data also suggested that SUV3 functions in splicing and the degradation of excised introns (i.e., null mutations in the degradosome components result in overaccumulation of intronic sequences and a reduction in mRNA levels), although the functions of the protein seem to be primarily associated with global mtRNA turnover and surveillance [73,74]. Defects in the organellar degradosomes led to defects in the processing of structural RNAs (e.g., rRNA and tRNAs), which resulted in altered organellar translation and mitogenome instability. It is hypothesized that the degradation of the excised organellar introns may enable the recycling of lowly-expressed splicing cofactors, which are typically tightly associated with their intron RNA ligands.

MSS116 is a model DEAD-box helicase, which contains the canonical tetrapeptide motif. The functions of the MSS116 protein are mainly linked to organellar splicing (Table 1). The *MSS116* gene was initially identified in a genetic screen for factors that regulate the splicing of organellar introns in yeast [75]. Experimental data further indicated that MSS116 assists in the splicing of virtually all the group I and II introns in yeast mitochondria [45]. MSS116 was also found to affect organellar translation, as indicated in mutant lines of yeast strains lacking mitochondrial introns, where the most notable reductions in protein levels were apparent in the cases of Cox1 and Cox3 [75]. Notably, the analysis of MSS116 sheds important light on the functions of RNA helicases in intron RNA splicing. In the group I ai5β intron, MSS116 was shown to facilitate a conformational change in the intron structure, following the first transesterification step (i.e., the release of the 5′ exon) that is necessary for the exon’s ligation reaction [76]. Also, MSS116 was found to assist in the folding of the group II ai2 intron, together with the ai2 maturase factor, into its catalytically active form [45]. These activities could relate to the roles of RNA helicases as either RNA chaperons, i.e., by unwinding undesirable structure intermediates (kinetic traps) of short duplexes [77,78], or alternatively may assist in the folding of the RNA by binding and stabilizing specific secondary and/or tertiary RNA base interactions (‘on-pathway intermediates’), in an ATP-independent manner [79]. Analysis of the 3D structure and biochemical assays indicated that the MSS116’s helicase core cooperates with its C-terminal domain to facilitate the intron splicing [80,81]. These analyses suggested that the C-terminal region of MSS116 has two distinct roles, by assisting in RNA binding as well as in stabilizing the helicase core. Remarkably, the functions of MSS116 in ai5γ intron splicing can be complemented by some other RNA helicases, such as the mitochondrial CYT-19 of *Neurospora crassa*, or the cytoplasmic DED1 helicase in yeast, whose functions are related to translation initiation [45,82]. The roles of plant helicases in mtRNA metabolism are less clear and require further investigation. Here, we summarize the current information about the roles of RNA helicases in group II intron splicing.

The nuclear genomes of land plants seem to harbor a large number of genes that encode DExH/D RNA helicases, as compared with other organisms [83]. More than 100 SF2 DNA or RNA helicase genes were previously described in the nuclear genome of the model angiosperms *Arabidopsis thaliana* and rice (*Oryza sativa*) [83,84]. The majority of the genes seem to be constitutively expressed in the plant and are likely to encode housekeeping proteins that are required for the maintenance of basic cellular functions. Although these are expected to have important roles in DNA or RNA metabolism, the specific functions of the majority of these enzymes are currently unknown.

**Figure 3:**
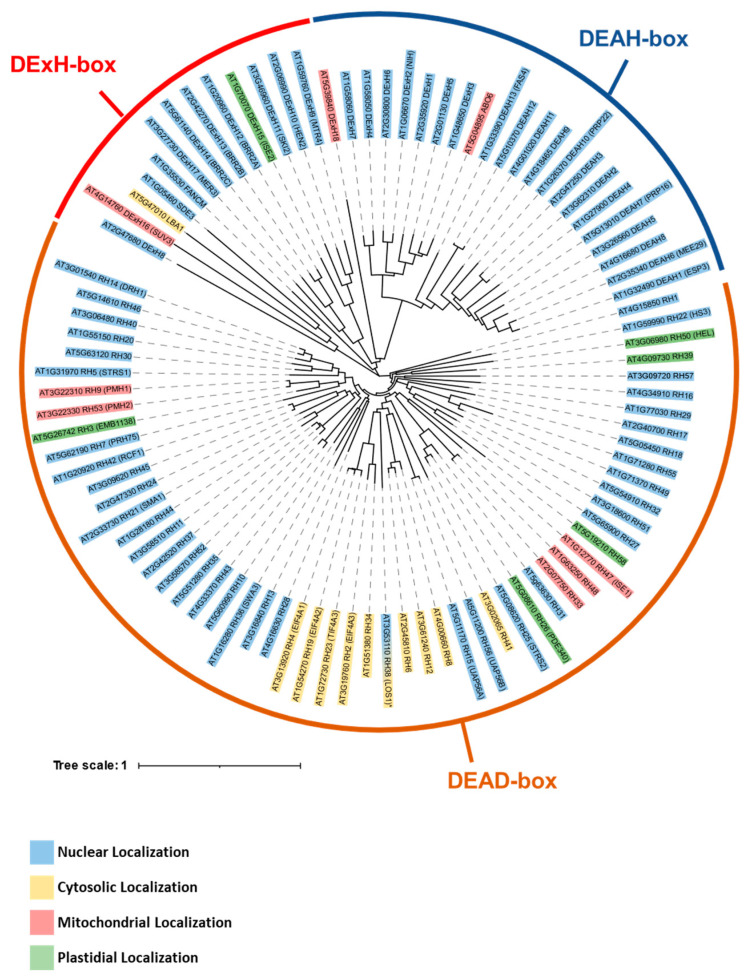
A phylogenetic analysis of the DExD/H box RNA helicases in *Arabidopsis thaliana*. The evolutionary relationships among different DExD/box helicases are illustrated by a phylogenetic tree. The tree was constructed by using the ‘Multiple Sequence Comparison by Log-Expectation’ (MUSCLE), based on the deduced protein sequences of homologous genes in *Arabidopsis thaliana*. The ClustalW algorithm was used for the alignment of the proteins and the tree was built using a neighbor-joining algorithm based on this alignment. The three RNA helicase groups, DExH, DEAD and DEAH box, are indicated in the figure. The tree distances represent the degree of divergence in the amino acid sequence between each branch in the tree. The predicted cellular localization of the gene products, as assigned by the ‘subcellular localization database for Arabidopsis proteins’ (SUBA5) database [57], are indicated. Blue for nuclear proteins, yellow for cytosolic, red for mitochondria and green for plastid/chloroplast proteins.

The Arabidopsis Information Resource (TAIR) and the UniProt databases suggest the presence of numerous genes encoding DNA or RNA helicases, with 92 of them being postulated to function as RNA helicases (Figure 3, Supplementary Table S1). Similar to previous reports [83,84], our phylogenetic analysis also indicates that the plant RNA helicases are clustered into 3 main subgroups, which include DEAH, DExH and DEAD-box RNA helicase family proteins (Figure 3). At least nine of the RNA helicases genes encode protein cofactors, which are predicted or shown to reside within the mitochondria in Arabidopsis (Table 1, Figure 3). These include a homolog of the yeast SUV3 protein that is also identified in rice, however, the specific roles of this factor in plant organellar RNA (or DNA) metabolism await further characterization (Table 1 and [53,54,55]). Notably, three specific RNA helicases (i.e., ABO6, PMH2 and RH33), which were also subjected to detailed analysis, were all shown to be involved in mitochondrial group II introns splicing.

*PUTATIVE MITOCHONDRIAL RNA HELICASE 1* (*PMH1*) and *PMH2* are situated very close to one another within chromosome 3 (Table 1). These also share a high sequence similarity and are likely representing a recent gene duplication event [85]. The PMH1 and PMH2 proteins harbor a DEAD-box helicase subdomain and an N-terminal region that is related to plant mitochondria localization signals. Accordingly, both PMH1 and PMH2 are identified in high molecular weight ribonucleoprotein (RNP) complexes in Arabidopsis mitochondria [50]. The *PMH1* gene is expressed at low levels in a tissue-specific manner (mainly in the flowers) and seems to be induced by cold stress, while *PMH2* likely encodes a housekeeping factor that is expressed in all tissues throughout plant growth and development. PMH2 was found to function as a general splicing factor, influencing the processing of numerous group II introns in Arabidopsis mitochondria [35,51]. Notably, the plant maturase factor (nMAT2; [86]) and PMH2 function in the splicing of a similar subset of group II introns [35,51]. It is, therefore, tempting to speculate that similarly to the roles of MSS116 in yeast, PMH2 may also function as an RNA chaperone that assists in the folding of maturase-bound pre-RNA transcripts in Arabidopsis mitochondria. Yet, much less is known about the roles of the PMH1 paralog in organellar RNA metabolism and gene expression. Based on its high sequence similarity with PMH2, and the lack of molecular phenotypes of *PMH1* knockout mutant lines, it is plausible that PMH1 may possess some functional redundancies with those of PMH2, especially in the flower tissues or under specific growth conditions, where the expression of the gene is upregulated [50].

*ABA OVERLY SENSITIVE 6* (*ABO6*) encodes a member of the DExH box helicase subfamily, which was identified in a genetic screen for ABA-mediated inhibition of primary root growth [48]. A point mutation in the sixth exon of *ABO6* gene-locus leads to reduced splicing efficiency of several mitochondrial pre-RNAs encoding the respiratory complex I Nad1, Nad2, Nad4 and Nad5 subunits. The point mutation (Gly-334 to Glu-334) in *abo6-1* mutant is located within the DExH motif of ABO6, in which the Gly-334 is identified as a well-preserved amino acid in ABO6 orthologs in different plant species. The functions of ABO6 seem to be essential during embryogenesis and early plant development, as heterozygous T-DNA insertional lines found within the coding region of the *AT5G04895* locus are unable to produce homozygous progeny. The growth and developmental defect phenotypes, and reduced levels of various organellar *nad* mRNA transcripts in *abo6* mutants, are likely associated with altered respiratory complex I (CI) biogenesis and function [19,87,88,89]. The accumulation of holo-CI (or partially-assembled CI intermediates) in *abo6* mutant lines has not been analyzed yet, and no data currently exist regarding the respiratory functions of the mutants versus the wild-type plants. However, the mutants are anticipated to exhibit altered OXPHOS functions, as the *abo6* mutants show an induction of the alternative respiratory pathway (i.e., alternative oxidases and rotenone-insensitive NADH reductases) and increased ROS production [48].

The DEA(D/H)-box RNA helicase family protein, RH33 (encoded by the *At2g07750* gene locus) (Table 1 and Table S1), is another mitochondrial helicase, which based on preliminary data is associated with mitochondrial group II introns splicing in Arabidopsis plants (Supplementary Figures S1 and S2). Based on its N-terminal region, the RH33 protein product is predicted to be localized to the mitochondria (Table 1 and Table S1; [57]). To establish the putative roles of RH33 in mitochondrial RNA (mtRNA) metabolism, we analyzed the growth phenotypes and RNA profiles of three individual T-DNA insertional lines within the *AT2G07750* gene locus, i.e., Sail_604_A01 (*rh33-1*) (found within the 5′-UTR, 16 nucleotides upstream to the AUG site), Salk_119034 (*rh33-2*) (located 221 nts upstream to the AUG site within the 5′-UTR) and Salk_119725 (*rh33-3*; found in the coding region, inside the exon 7 region) (Figure S1A). The *rh33-1* mutants exhibited normal growth phenotypes when grown on MS-agar plats, under optimal growth conditions (i.e., at 22 °C), but showed retarded growth phenotypes, and more notably altered root morphology, when grown at 28 °C (Figure S1B). An association between mtRNA processing defects and altered root development was recently indicated for a few temperature-sensitive mutants encoding mitochondrial RBPs, i.e., the RRD1 a ribonuclease (altered mRNA deadenylation), and two PPR editing factors, RRD2 and RID4 [90], as well as the *rpd1* mutants [91,92,93], which are affected in the expression of the PORR-family splicing cofactor, ROOT PRIMORDIUM DEFECTIVE 1 [93]. Yet, the phenotypes of the homozygous *rh33-2* mutant were similar to those of the wild type (Col-0) plants, while no homozygous plants could be established for the *rh33-3* line that contains a T-DNA insertion within the 7th exon of *RH33* gene (Figure S1A), suggesting that the functions of RH33 are essential during embryogenesis and for normal seed development. A primary analysis of the RNA profiles of *rh33* plants indicated reduced mRNA accumulation of various *nad1* and *nad7* transcripts in *rh33-1* plants, whose coding regions are both interrupted by several group II introns (Figure S1C). However, the RNA profiles of *rh33-2* plants (containing a T-DNA insertion in the 5′ UTR) were similar to those of the wild-type plants, possibly because the insertion within the 5′ UTR of *rh33-2* has not affected the expression of the gene [94]. We further assumed that the reduced mRNA levels we see in *nad1* exons 1-2, 2-3, 3-4 and 4-5, and *nad7* exons 2-3 in *rh33-1*, may correspond to defects in the excision of the introns that reside within the three mitochondrial *nad* genes. Indeed, RT-qPCRs further indicated splicing defects (i.e., accumulation of pre-RNAs that are correlated with reduced levels of their corresponding mRNAs) in the *rh33-1* mutant (Figure S2). These data support a role for RH33 in the splicing of various group II-type introns in Arabidopsis mitochondria. The intracellular location(s) and the effects of altered RH33 expression on the respiratory functions, OXPHOS complex assembly and organellar biogenesis are currently analyzed.

Analysis of a mitochondria-localized RNA helicase in corn (*Zea mays*), termed RH48 (Table 1 and Table S1), indicates that the protein facilitates the processing of several organellar group II introns, including the second intron in *nad2* (i.e., *nad2* i2), *nad5* i1, *nad7* introns 1, 2 and 3, and the single intron within the *ccmFc* pre-RNA transcript [52]. The *Zm-rh48* mutants displayed seed and embryo developmental defect phenotypes, which are postulated to be associated with altered complex I and cytochrome C biogenesis [52]. Two-hybrid screens indicated that RH48 interacts with several known splicing factors, including PPR-SMR1 and SPR2 [52]. ZmRH48 may also play a role in the C-to-U deamination (i.e., RNA editing) of C-515 of the *atp6* transcript [52]. Yet, its function in the editing *atp6*-515 is less pronounced, which may indicate a pleiotropic effect due to the altered mtRNA metabolism [32,95,96,97,98]. Arabidopsis contains a homolog of *ZmRH48* (AtRH48, At1G63250), but its roles in organellar gene expression remain to be addressed.

Another DEAD-Box RNA helicase that is localized to the mitochondria is the INCREASED SIZE EXCLUSION LIMIT 1 (ISE1) protein (Table 1; [49]). Similar to the functions of several other mtRNA processing enzymes, [2,3,19,87], the functions of ISE1 are essential for normal embryogenesis. The *ise1* mutants further show altered plasmodesmata (PD) structure and increased PD-mediated transport of fluorescent tracers, which may relate to altered cellular metabolism [49]. Similarly, reduced expression of *ISE1* in tobacco (*Nicotiana benthamiana*) cells also leads to increased intercellular movement of a GFP-TMV-MP fusion protein through the PD [49].

In addition to these factors, Arabidopsis plants encode a homolog (i.e., AT4G14760, AtSUV3) of the OsSUV3 protein, which was found to possess RNA unwinding activity and to provide salt tolerance in transgenic over-expressed lines [53,55]. Due to their high homology with the yeast SUV3 protein [46,47], it has been proposed that the plant orthologs also play a role in the mitochondrial degradosome, but such hypotheses need to be experimentally supported. No data currently exist for the DExH18 protein, which is encoded by the *AT5G39840* gene-locus in Arabidopsis and predicted to be localized to the mitochondria (Table 1). The phylogenetic data indicate that AT5G39840 is clustered together with various DExH helicase proteins (Figure 3).

## 5. Conclusions and Perspectives

RNA helicases are key components in complex cellular RNA metabolism activities. These factors also play pivotal roles in maintaining mtDNA maintenance and in organellar gene expression. Their unwinding activity is fundamental for the proper maturation and functioning of numerous RNA molecules, thereby contributing to the regulation of gene expression (transcription, post-transcriptional RNA processing and translation) and overall cellular homeostasis. Ongoing research continues to uncover the specific roles and mechanisms of different RNA helicases in cellular, nuclear and organellar RNA metabolism. As indicated above, the canonical group I and II introns are catalytic RNAs that are able to catalyze their own excision in vitro (usually under non-physiological conditions) [26,32,33,34,99,100]. However, for their efficient splicing in vivo, all group I or II introns rely on the activities of various proteinaceous cofactors. In yeast and plant mitochondria, these include maturases, as well as various nuclear-encoded RNA-binding proteins, which function on specific introns or may generally affect the splicing of several or many pre-RNAs, see e.g, [21,35,37,38,86,101,102,103,104]. RNA helicases were shown to be required for an efficient splicing of all the mitochondrial group I and II introns, including those that do not require the assistance of an intron-encoded maturase.

## Figures and Tables

**Table 1:** Mitochondrial RNA helicases in fungi and plants.

RNA Helicase	Gene I.D.	Organism *^1^	Specific Role(s) in mt-RNA Metabolism	REFs
IRC3	S000002740	*S.c.*	mtDNA maintenance or stability	[43]
MRH4	S000003032	*S.c.*	Translation, ribosome biogenesis	[44]
MSS116	S000002602	*S.c.*	Splicing (group I and II introns)	[45]
SUV3	S000005950	*S.c.*	Nucleolysis (degredosomal factor)	[46,47]
ABO6	At5g04895	*A.t.*	Splicing (group II introns)	[48]
ISE1/EMB1586	At1g12770	*A.t.*	T.B.D. *^2^, affects mitochondria biogenesis	[49]
DExH18	At5g39840	*A.t.*	T.B.D., predicted to the mitochondria *^3^	---
PMH1	At3g22310	*A.t.*	T.B.D., found in large RNP complexes *^4^	[50]
PMH2	At3g22330	*A.t.*	Splicing (group II introns)	[35,50,51]
RH33	At2g07750	*A.t.*	Splicing (group II introns)	**This study**
RH48	At1g63250	*A.t.*	T.B.D., homolog of Zm-RH48	---
RH48	GRMZM2G171801	*Z.m.*	Splicing (group II introns)	[52]
SUV3 (At)	At4g14790	*A.t.*	T.B.D., RNA metabolisms	[53]
SUV3 (Os)	GQ982584	*O.s.*	T.B.D., DNA/RNA metabolisms	[54,55]
RECG1	At2g01440	*A.t.*	T.B.D., mtDNA and cpDNA maintenance	[56]

*^1^—*A.t.*, *Arabidopsis thaliana*; *O.s.*, *Oryza sativa*; *S.c.*, *Saccharomyces cerevisiae*; *Z.m.*, *Zea mays.* *^2^—T.B.D., To be determined, awaiting further analysis. *^3^—Predicted mitochondria localization [57,58]. *^4^—RNP complexes, ribonucleoprotein complexes.

## Data Availability

Not applicable.

## Supplementary Materials

The following supporting information can be downloaded at: https://www.mdpi.com/article/10.3390/ijms25105502/s1.
