# Supplementary material for: Mitochondrial RNA Helicases: Key Players in the Regulation of Plant Organellar RNA Splicing and Gene Expression
Source: Int J Mol Sci. 2024 May 17;25(10):5502. doi: 10.3390/ijms25105502 (PMC11122041; doi:10.3390/ijms25105502)
Supplement: Supplementary file 1 [file ijms-25-05502-s001.zip › ijms-2936998-supplementary.pdf]

Supplementary Table S1. RNA helicases encoded in *Arabidopsis thaliana* genome.

Gene I.D. *[1]	Protein I.D. / Gene function	Predicted loci*[2]
Putative RNA helicases		
At1g05460	RNA helicases SDE3, Post-transcriptional gene silencing	Nuc
At1g06670	Nucleuslear DEIH-box helicase (NIH)	Nuc
At1g08600	ATRX/CHR20	Nuc
At1g11100	SNF2 domain-containing protein	Nuc
At1g12770	RH47, Regulating cell-to-cell transport via plasmodesmata	Mit
At1g16280	DEA(D/H)-box RNA helicase, RH36	Nuc
At1g16970	KU70 homolog, Maintenance of the telomeric C strand	Nuc
At1g20920	DEAD box RNA helicase (RH42)	Nuc
At1g20960	EMB1507, U5 small Nucleuslear riboNucleusleoprotein helicase, putative	Nuc
At1g31970	Stress response suppressor 1 (STRS1, RH5)	Nuc
At1g32490	Encodes a homolog of the yeast PRP2 protein, RNA splicing	Nuc
At1g35530	DEA(D/H)-box RNA helicase family protein	Nuc
At1g48650	DEA(D/H)-box RNA helicase family protein	Mit
At1g48920	Nucleusleolin like 1 (AtNUCLEUS-L1), rRNA processing, ribosome biosynthesis	Nuc
At1g51380	Putative translation initiation factor 4A (RH2)	Cyt
At1g55150	DEA(D/H)-box RNA helicase, putative (RH20)	Nuc
At1g59760	RNA helicase, ATP-dependent, SK12/DOB1 protein	Nuc
At1g59990	DEA(D/H)-box RNA helicase, putative (RH22)	Clp
At1g61140	SNF2 domain-containing protein, EDA16 (EMB-16)	Nuc
At1g63250	DEA(D/H)-box RNA helicase family protein (RH48), putative splicing factor	Mit
At1g71280	DEA(D/H)-box RNA helicase family protein (RH55)	Nuc
At1g72730	DEA(D/H)-box RNA helicase protein, Eukaryote translation initiation factor 4A	Nuc
At1g77050	DEA(D/H)-box RNA helicase family protein (RH29)	Mit
At1g79890	RAD3-like DNA-binding helicase protein	Nuc
At1g79950	RAD3-like DNA-binding helicase protein	Nuc
At2g01130	DEA(D/H)-box RNA helicase family protein	Nuc
At2g01440	DEA(D/H)-box RNA helicase family protein	Mit
At2g06990	SK12/DOB1 protein, a putative DExH-box RNA helicase	Nuc
At2g07750	DEA(D/H)-box RNA helicase family protein (RH33), group II intron splicing*[3]	Mit
At2g33730	P-loop containing NTP hydrolases superfamily protein (RH21)	Nuc
At2g35920	RNA helicase family protein	Nuc
At2g40700	RNA helicase family protein (RH17)	Nuc
At2g42270	U5 small nuclear ribonucleoprotein helicase	Nuc
At2g42520	P-loop containing NTP hydrolases superfamily protein (RH37)	Nuc
At2g45810	DEA(D/H)-box RNA helicase family protein (RH6)	Cyt
At2g47250	RNA helicase family protein	Nuc
At2g47330	P-loop containing NTP hydrolases superfamily protein (RH24)	Nuc
At3g01540	DEAD-box RNA helicase, DRH1	Nuc
At3g02060	DEA(D/H)-box RNA helicase family protein	Clp
At3g06480	DEAD box RNA helicase family protein (RH40)	Nuc
At3g06980	DEA(D/H)-box RNA helicase family protein (RH50)	Clp
At3g09620	P-loop containing NTP hydrolases superfamily protein (RH45)	Nuc

At3g09720	P-loop containing NTP hydrolases superfamily protein (RH57)	Nuc
At3g13920	Eukaryotic translation initiation factor 4A1; (RH4)	Cyt
At3g16600	SNF2 domain-containing protein	Nuc
At3g16840	P-loop containing NTP hydrolases superfamily protein (RH13)	Nuc
At3g18600	P-loop containing NTP hydrolases superfamily protein (RH51)	Nuc
At3g22310	Putative mitochondrial RNA helicase 1 (PMH1, RH9)	Mit
At3g46960	RNA helicase SK12/DOB1 protein, HUA enhancer 2 (HEN2)	Nuc
At3g53110	P-loop containing NTP hydrolases superfamily protein (LOS4)	Nuc/Cyt
At3g58510	DEA(D/H)-box RNA helicase family protein; (RH11)	Nuc
At3g61240	DEA(D/H)-box RNA helicase family protein (RH12)	Nuc
At3g62310	RNA helicase family protein;	Nuc
At4g00660	RNAhelicase-like 8; (RH8)	Nuc
At4g01020	helicase domain-containing protein / IBR domain-containing protein	Nuc
At4g09730	Introduction of the hidden break into the 23S rRNA in the chloroplasts (RH39)	Clp
At4g15850	RNA helicase 1; plant DEAD box-like RNA helicase.	Nuc
At4g16630	DEA(D/H)-box RNA helicase family protein (RH28)	Nuc
At4g16680	P-loop containing NTP hydrolases superfamily protein, similar to MEE29	Nuc
At4g18465	RNA helicase family protein (EMB-2733)	Nuc
At4g25120	Encodes a homolog of the yeast SRS2 helicase	Nuc
At4g33370	DEA(D/H)-box RNA helicase family protein; (RH43)	Nuc
At4g34910	P-loop containing NTP hydrolases superfamily protein (RH16)	Nuc
At4g35740	DEA(D/H)-box RNA helicase RECQL3	Nuc
At5g05130	DNA/RNA helicase protein (SNF2 domain-containing protein)	Nuc
At5g05450	P-loop containing NTP hydrolases superfamily protein (RH18)	Nuc
At5g07810	SNF2 domain-containing protein / HNH endonuclease domain-containing protein	Nuc
At5g08610	P-loop containing NTP hydrolases superfamily protein (RH26)	Clp
At5g08620	Stress response suppressor 2 (STRS2, RH25)	Nuc
At5g10370	Helicase domain-containing protein / IBR domain-containing protein	Nuc
At5g11170	DEA(D/H)-box RNA helicase family protein (RH15)	Nuc
At5g11200	DEA(D/H)-box RNA helicase family protein (RH56)	Nuc
At5g14610	DEAD box RNA helicase family protein (RH46)	Nuc
At5g19210	P-loop containing NTP hydrolases superfamily protein (RH58)	POs
At5g41360	Homolog of Xeroderma pigmentosum complementation group B 2 (XPB2)	Nuc
At5g41370	Homolog of Xeroderma pigmentosum complementation group B 2 (XPB1)	Nuc
At5g51280	DEAD-box protein abstrakt, putative; (RH35)	Nuc
At5g54910	DEA(D/H)-box RNA helicase family protein; (RH32)	Nuc
At5g60990	DEA(D/H)-box RNA helicase family protein; (RH10)	Nuc
At5g62190	DEA(D/H)-box RNA helicase family protein; (PRH75)	Nuc
At5g63120	P-loop containing NTP hydrolases superfamily protein; ethylene-responsive (RH30)	Nuc
At5g63630	P-loop containing NTP hydrolases superfamily protein; (RH31)	Nuc
At5g65900	DEA(D/H)-box RNA helicase family protein; (RH27)	Nuc
Putative DNA helicases		
At1g08840	EMB2411, putative DNA replication helicase	Nuc
At1g20750	RAD3-like DNA-binding helicase protein	Nuc
At1g48310	Chromatin remodeling factor18 (CRF18)	Nuc
At1g60930	RECQ helicase L4B (AtRECQ4B), Involved in homologous recombination	Mit
At2g07690	Minichromosome maintenance (MCM) family protein, DNA replication initiation	Nuc

At2g14050	Minichromosome maintenance 9; MCM9	Nuc
At2g16440	Minichromosome maintenance (MCM) family protein, DNA replication initiation	Nuc
At2g20980	Minichromosome maintenance 10, MCM10	Nuc
At3g05740	RECQ helicase l1 (RECQI1), DNA recombinantion	Nuc
At3g09660	Minichromosome maintenance 8 (MCM8)	Nuc
At4g02060	Minichromosome maintenance family protein MCM7	Nuc
At1g48050	KU80 family protein, Mutants are defective in T-DNA integration	Nuc
At1g31360	RECQ helicase L2 (RECQL2), DNA unwinding	Nuc
At5g20420	Chromatin remodeling 42 (CHR42)	Nuc
At5g46280	Minichromosome maintenance family protein MCM3	Nuc
At5g63950	chromatin remodeling 24	Nuc
At1g03750	Chromatin remodeling 9; switch 2;	Nuc
At1g10930	DNA helicase RECQl4A; (AtRECQ4A)	Nuc
At1g05490	Chromatin remodeling 31	Nuc
At1g27880	DEA(D/H)-box RNA helicase family protein; Similar to RecQl3 (Recq-like 3)	Nuc
At2g02090	SNF2 domain-containing protein; Chromatin remodeling 19	Nuc
At2g03270	DNA-binding protein, putative;	Nuc
At2g30800	RNA or DNA helicase (ATVT1); expressed specifically in tapetum and vascular tissue	Nuc
At2g13370	Chromatin remodeling 5	Nuc
At3g27730	DNA helicase (MER3); required for interference-sensitive meiotic crossover events	Nuc
At3g51690	DNA helicase homolog PIF1; PIF1 helicase	Nuc
At3g54280	DNA binding; ATP binding; nucleic acid binding helicase	Nuc
At5g22330	P-loop containing NTP hydrolases superfamily protein (AtTIP49A)	Nuc
At5g27680	RECQ helicase SIM; DNA helicase (RECQSIM)	Nuc
At5g67630	P-loop containing NTP hydrolases superfamily protein	Nuc

*[1] – Gene i.d. according to 'The Arabidopsis Information Resource' (TAIR) database.
*[2] – Arabidopsis protein subcellular location database, SUBA (https://suba.live/); Clp – chloroplast/plastid, Cyt – cytosol, Mit – mitochondria, Nuc – nucleus, POs – peroxisome.
*[3] – This study.

Supplementary Figure legends

Figure S1. The RNA helicase RH33 affects the processing of various protein-encoding transcripts in Arabidopsis mitochondria.

(A) Scheme of the RNA Helicase 33 (RH33), encoded by the At2g07750 gene locus. Black boxes represent exons, lines indicate to intron regions, while grey boxes show the 5′ and 3′ UTRs. The different T-DNA insertions are indicated above the gene structure as triangles.

(B) Growth and developmental phenotypes associated with the *rh33* mutants. Seeds of wild type (Col-0) and the three T-DNA insertional lines, *rh33-1* (Sail_604_A01), *rh33-2* (Salk_119034), and *rh33-3* (Salk_119725), were germinated on MS-agar plates supplemented with 1% sucrose. The picture shows 3-week-old seedlings grown under optimal growth conditions (i.e., short-day conditions, 10:14 light to dark, with a light intensity of ~150 $\mu E \cdot m^{-2} \cdot s^{-1}$, with 50% relative humidity (RH), under either 22°C (optimal condition) or 28°C (restrictive condition).

(C) Transcript abundance in *rh33* mutants. Real-time quantitative reverse transcription polymerase chain reaction (RT-qPCR) analyses of mitochondrial genes. Total RNA was extracted from 3-week-old seedlings of Col-0 and mutant plants were reverse-transcribed, and the relative steady-state levels of cDNAs corresponding to the different organellar transcripts were evaluated by qPCR with primers that specifically amplified mtRNAs. Specific oligonucleotides used in the RT-qPCRs are indicated in Ref's [1, 2].

Figure S2. Analysis of the splicing efficiencies of mitochondrial group II-type introns in *rh33-1* mutant.

The splicing efficiencies of the 23 group-II introns in Arabidopsis wild type (Col-0) and *rh33-1* mutant plants were evaluated by RT-qPCR, with RNA extracted from *3*-week-old wild-type (Col-0) and *rh33-1* mutant plants. The splicing efficiencies were estimated from the relative accumulation (pre-RNA/mRNA ratio's) of organellar transcripts in the mutant line versus the wild type plants by RT-qPCR with specific oligonucleotides designed to intron-exon regions (pre-RNAs) and exon-exon (mRNAs). Specific oligonucleotides used in the RT-qPCRs are indicated in Ref's [1, 2].

References

1. Best, C.; Mizrahi, R.; Edris, R.; Tang, H.; Zer, H.; Colas des Francs-Small, C.; Finkel, O. M.; Zhu, H.; Small, I. D.; Ostersetzer-Biran, O., MSP1 encodes an essential RNA-binding pentatricopeptide repeat factor required for nad1 maturation and complex I biogenesis in Arabidopsis mitochondria. *New Phytol* **2023,** 238, (6), 2375-2392.
2. Shevtsov-Tal, S.; Best, C.; Matan, R.; Chandran, S. A.; Brown, G. G.; Ostersetzer-Biran, O., nMAT3 is an essential maturase splicing factor required for holo-complex I biogenesis and embryo development in *Arabidopsis thaliana* plants. *Plant J* **2021,** 106, (4), 1128-1147.

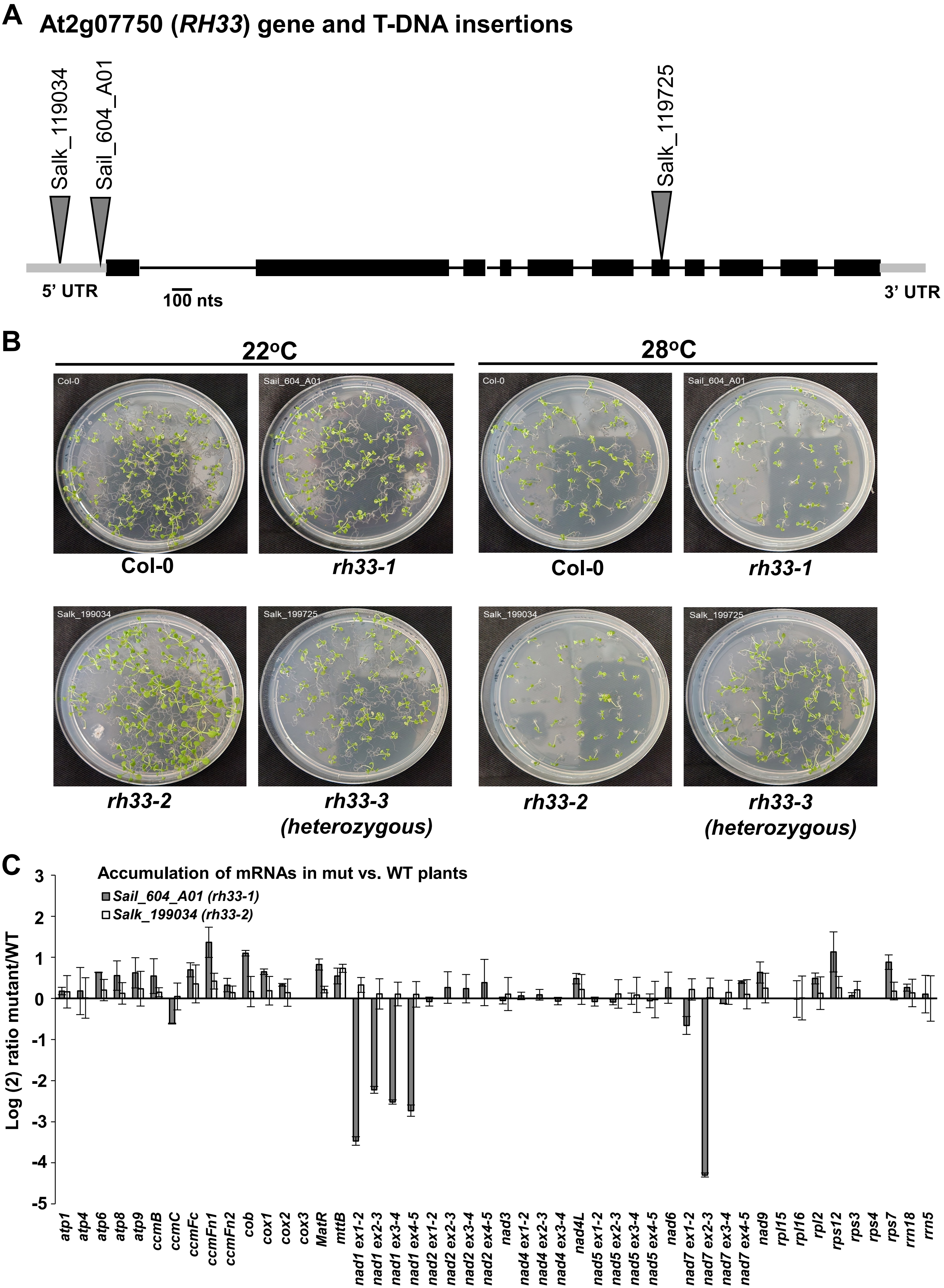

Supplementary Fig. S1. The RNA helicase RH33 affects the processing of various protein-encoding transcripts in Arabidopsis mitochondria.

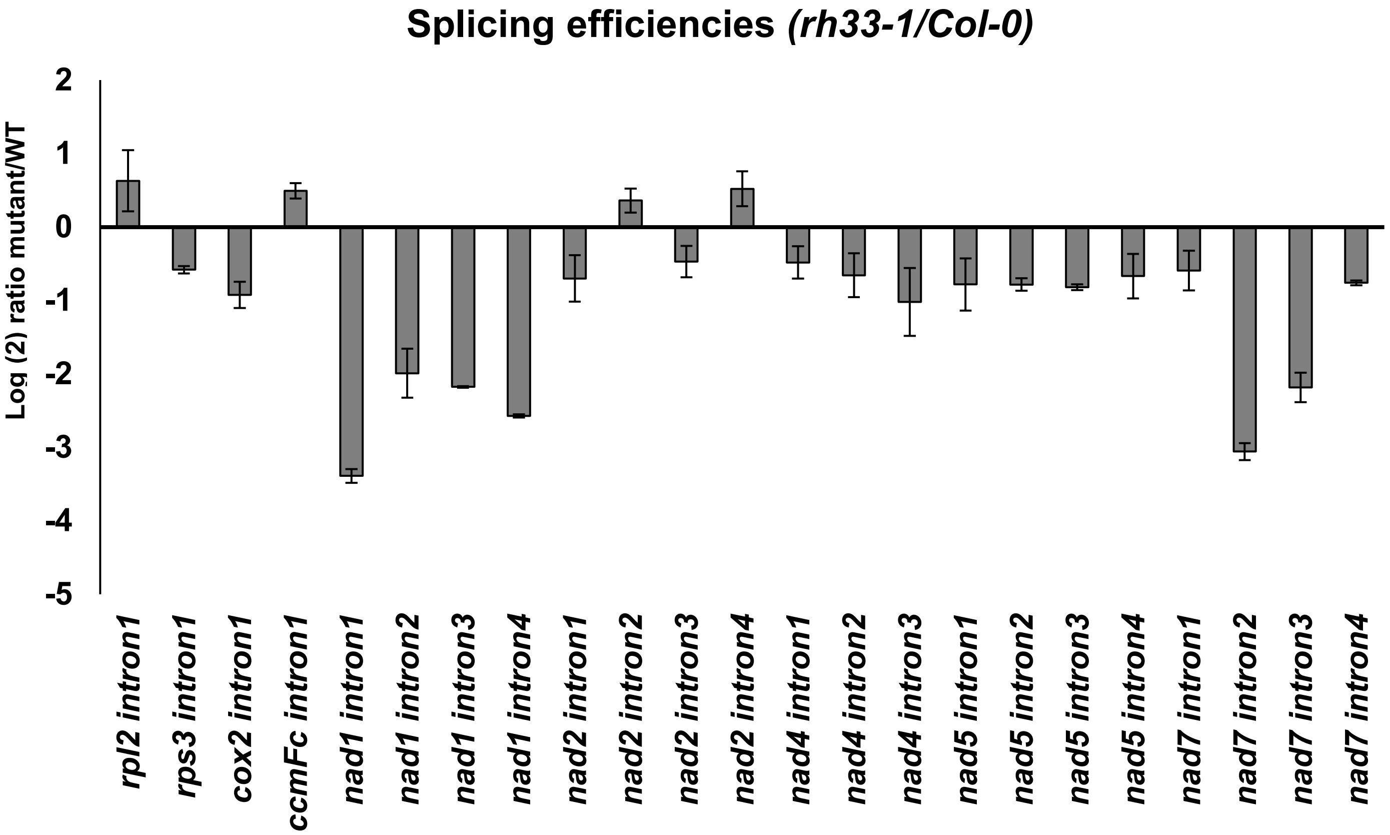

Supplementary Fig. S2. Analysis of the splicing efficiencies of mitochondrial group II-type introns in *rh33-1* mutant.
